# Opioid overdose decedent characteristics during COVID-19

**DOI:** 10.1080/07853890.2022.2067350

**Published:** 2022-04-25

**Authors:** Gian-Gabriel P. Garcia, Erin J. Stringfellow, Catherine DiGennaro, Nicole Poellinger, Jaden Wood, Sarah Wakeman, Mohammad S. Jalali

**Affiliations:** a Georgia Institute of Technology, Atlanta, GA, USA; b Massachusetts General Hospital, Harvard Medical School, Boston, MA, USA

**Keywords:** Opioid overdoses, COVID-19, decedent characteristics

## Abstract

**Introduction:**

Alongside the emergence of COVID-19 in the United States, several reports highlighted increasing rates of opioid overdose from preliminary data. Yet, little is known about how state-level opioid overdose death trends and decedent characteristics have evolved using official death records.

**Methods:**

We requested vital statistics data from 2018–2020 from all 50 states and the District of Columbia, receiving data from 14 states. Accounting for COVID-19, we excluded states without data past March 2020, leaving 11 states for analysis. We defined state-specific analysis periods from March 13 until the latest reliable date in each state’s data, then conducted retrospective year-over-year analyses comparing opioid-related overdose death rates, the presence of specific opioids and other psychoactive substances, and decedents’ sex, race, and age from 2020 to 2019 and 2019 to 2018 within each state’s analysis period. We assessed whether significant changes in 2020 vs. 2019 in opioid overdose deaths were new or continuing trends using joinpoint regression.

**Results:**

We found significant increases in opioid-related overdose death rates in Alaska (55.3%), Colorado (80.2%), Indiana (40.1%), Nevada (50.0%), North Carolina (30.5%), Rhode Island (29.6%), and Virginia (66.4%) – all continuing previous trends. Increases in synthetic opioid-involved overdose deaths were new in Alaska (136.5%), Indiana (27.6%), and Virginia (16.5%), whilst continuing in Colorado (44.4%), Connecticut (3.6%), Nevada (75.0%), and North Carolina (14.6%). We found new increases in male decedents in Indiana (12.0%), and continuing increases in Colorado (15.2%). We also found continuing increases in Black non-Hispanic decedents in Massachusetts (43.9%) and Virginia (33.7%).

**Conclusion:**

This research analyzes vital statistics data from 11 states, highlighting new trends in opioid overdose deaths and decedent characteristics across 10 of these states. These findings can inform state-specific public health interventions and highlight the need for timely and comprehensive fatal opioid overdose data, especially amidst concurrent crises such as COVID-19.

**Key messages:**
- Our results highlight shifts in opioid overdose trends during the COVID-19 pandemic that cannot otherwise be extracted from aggregated or provisional opioid overdose death data such as those published by the Centres for Disease Control and Prevention.
- Fentanyl and other synthetic opioids continue to drive increases in fatal overdoses, making it difficult to separate these trends from any possible COVID-19-related factors.
- Black non-Hispanic people are making up an increasing proportion of opioid overdose deaths in some states.
- State-specific limitations and variations in data-reporting for vital statistics make it challenging to acquire and analyse up-to-date data on opioid-related overdose deaths. More timely and comprehensive data are needed to generate broader insights on the nature of the intersecting opioid and COVID-19 crises

## 1. Introduction

Since COVID-19 emerged in the United States, experts warned that the pandemic’s strain on healthcare systems, economic stability, and social support structures would threaten many vulnerable individuals’ physical and mental well-being [1,2]. Concurrently, the opioid overdose crisis has continued to evolve, becoming more fatal since the onset of COVID-19.

Early studies reported increases in opioid overdose admissions and deaths across emergency departments in San Francisco from January 1, 2020 to April 18, 2020 [3], Indianapolis from March 25, 2019 to July 24, 2020 [4], and Kentucky, Massachusetts, New York, and Ohio from January 1, 2020 to August 1, 2020 [5,6]. Official cause of death data from Los Angeles County from January 1, 2019 to July 2020 [7] and Massachusetts from March 24, 2020 to November 8, 2020 [8] supported findings from these hospital data. Nationally, 2020 was the deadliest year for opioid overdose deaths on record [9], and moreover, recent provisional estimates from the United States Centres for Disease Control and Prevention (CDC) indicate that opioid overdose deaths have increased to more than 75,000 in the twelve-month period ending in June 2021 [10]. These estimates, along with recent increases in any drug overdose-related cardiac arrests nationwide [11], corroborate reports around the country linking increasing opioid overdose trends to COVID-19 [12].

This study extends previous analyses by: a) characterising shifting trends in opioid overdose deaths by substance, sex, age, and race; b) distinguishing between *new* trends (i.e. change from 2019–2020 was non-existent from 2018–2019) and *continuing* trends (i.e. change from 2019–2020 existed from 2018–2019); and c) analysing confirmed state-level mortality data rather than provisional data. We emphasise that our analysis includes detailed data not reported by the CDC (i.e. an expanded set of substances and decedent demographics). Moreover, the most recent data provided by the CDC are provisional estimates of overdose deaths, whereas our findings are drawn from confirmed deaths.

## 2. Materials and methods

### 2.1. Study data and variables

We submitted requests to all 50 states and the District of Columbia for vital statistics data from 2018–2020. Data were received from 14 states. Because we aimed to analyse changes in opioid overdose decedent characteristics during COVID-19, we only included states in our analysis that provided vital statistics data extending beyond March 2020. Hence, our analysis included eleven states: Alaska, Colorado, Connecticut, Indiana, Massachusetts, Nevada, North Carolina, Rhode Island, Utah, Virginia, and Wyoming. This study was deemed exempt from review by Mass General Brigham’s institutional review board.

### 2.2. Study variables

To facilitate our year-over-year analysis (i.e. comparing the same time period over 2018–2020), we separately defined an *analysis period* for each state. Each analysis period began on March 13 (i.e. when COVID-19 was declared a national emergency in the United States) until the latest reliable date of analysis as estimated, in part, by the state’s vital statistics experts. For Indiana, data were only available on a monthly basis and thus, its analysis period lasted from March 1 through June 30. These analysis periods differed across each state due to variations in each state’s death certification processes [13].

Within each state’s analysis period, we extracted opioid overdose death records from 2018–2020, i.e. records where ICD-10 codes T40.0, T40.1, T40.2, T40.4, and/or T40.6 were listed as a final or supporting cause of death [14]. Finally, we extracted each decedent’s age, sex, and race.

### 2.3. Statistical analysis

For 2018, 2019, and 2020 in each state’s analysis period, we computed annual opioid overdose death rates per 100,000 people and the proportion of opioid overdoses deaths in which opioid substances (i.e. ICD-10 codes T40.0, T40.1, T40.2, T40.4, and T40.6) or psychoactive substances (i.e. ICD-10 codes T40.5, T42.4, and T43.6) were involved. We then summarised decedents’ age (i.e. by age group in 10-year increments), sex (i.e. female, male, unknown), and race (i.e. Native American/Alaska Native/Other non-Hispanic, Asian non-Hispanic, Black non-Hispanic, Hispanic, White non-Hispanic, and unknown).

Next, we performed year-over-year analyses, comparing 2020 vs. 2019 and 2019 vs. 2018 in each state’s analysis period. For example, in Alaska, we compared data spanning March 13, 2020 to November 30, 2020 to data in the timeframe March 13, 2019 to November 30, 2019. We then compared data in the latter timeframe to data from March 13, 2018 to November 30, 2018. Specifically, we compared the mean annual opioid overdose death rates across different years using the bootstrap two-sample t-test [15] and the substances present and decedent demographics across different years using Pearson’s Chi-squared test. To determine which demographic categories were driving significant differences in age and race, we performed post-hoc Chi-squared analyses on expected residuals [16] using the Benjamini-Hochberg p-value correction for multiple comparisons [17].

Finally, we performed yearly trend analysis using joinpoint regression to confirm whether significant changes in 2020 vs. 2019 were *new* trends (i.e. model with 1 joinpoint is significantly different from model with 0 joinpoints) or *continuing* trends (i.e. model with 1 joinpoint is not significantly different from model with 0 joinpoints). All data analyses were performed using R version 4.1.0 and Joinpoint version 4.9.0.0.

## 3. Results

### 3.1. Opioid overdose death rates

Table 1 summarises each state’s analysis period and comparisons in annual opioid overdose deaths per 100,000 people plus overdose deaths by substance. The shortest analysis period was in Indiana (March 1 to June 30) and the longest analysis periods were in Connecticut, Massachusetts, and Utah (March 13 to December 31). Comparing 2020 to 2019 in each state’s analysis period, the annual overdose death rate per 100,000 people increased in Alaska (13.85 vs. 8.92, *p =* .020), Colorado (18.24 vs.10.12, *p <* .001), Indiana (25.98 vs. 18.54, *p =* .038), Nevada (20.01 vs. 13.34, *p* < .001), North Carolina (22.79 vs. 17.47, *p <* .001), Rhode Island (30.39 vs.23.45, *p =* .011), and Virginia (21.46 vs. 12.90, *p* < .001). Compared to 2019 vs. 2018, our joinpoint regression analysis reveals that all significant increases were continuing trends.

**Table 1.** Analysis of opioid overdose death rates and substances present in 2018–2020.

					Opioids^a^				**Other Psychoactive Substances** ^a^		
State	Analysis Period	Year	Total Opioid OD Deaths	Annual Opioid OD Deaths per 100,000 People	*T40.1: Heroin*	*T40.2: Natural and semi-synthetic opioids*	*T40.4: Synthetic opioids*	*T40.6: Unspecified narcotic*	*T40.5: Cocaine*	*T420.4: Benzodiazepine*	*T43.6: Psychostimulants*
Alaska	*March 13 - November 30*	2018	39	7.34	15 (38.5%)	21 (53.8%)	9 (23.1%)	19 (48.7%)	3 (7.7%)	11 (28.2%)	17 (43.6%)
		2019	47	8.92	28 (59.6%)	20 (42.6%)	12 (25.5%)	13 (27.7%)*	1 (2.1%)	8 (17.0%)	25 (53.2%)
		2020	73	13.85*	22 (30.1%)**	25 (34.2%)	44 (60.3%)***^†††^	19 (26.0%)	10 (13.7%)*^†††^	10 (13.7%)	32 (43.8%)
Colorado	*March 13 - August 30*	2018	246	9.22	104 (42.3%)	107 (43.5%)	53 (21.5%)	7 (2.8%)	31 (12.6%)	38 (15.4%)	64 (26.0%)
		2019	273	10.12	90 (33.0%)*	105 (38.5%)	113 (41.4%)***	10 (3.7%)	36 (13.2%)	38 (13.9%)	73 (26.7%)
		2020	492	18.24***	108 (22.0%)***	130 (26.4%)***	294 (59.8%)***	13 (2.6%)	94 (19.1%)*	62 (12.6%)	152 (30.9%)
Connecticut	*March 13 - December 31*	2018	782	27.17	279 (35.7%)	122 (15.6%)	647 (82.7%)	5 (0.6%)	233 (29.8%)	209 (26.7%)	41 (5.2%)
		2019	958	33.36***	288 (30.1%)*	174 (18.2%)	846 (88.3%)***	4 (0.4%)	298 (31.1%)	240 (25.1%)	50 (5.2%)
		2020	969	33.74	151 (15.6%)***^†††^	169 (17.4%)	887 (91.5%)*	2 (0.2%)	280 (28.9%)	222 (22.9%)	71 (7.3%)
Indiana^b^	*March 1 - June 30*	2018	385	17.26	117 (30.4%)	124 (32.2%)	238 (61.8%)	20 (5.2%)	60 (15.6%)	99 (25.7%)	80 (20.8%)
		2019	416	18.54	114 (27.4%)	122 (29.3%)	286 (68.8%)*	22 (5.3%)	66 (15.9%)	79 (19.0%)*	109 (26.2%)
		2020	583	25.98*	66 (11.3%)***	145 (24.9%)	512 (87.8%)***^†††^	17 (2.9%)	73 (12.5%)	79 (13.6%)*	147 (25.2%)
Massachusetts	*March 13 - December 31*	2018	1561	28.17	398 (25.5%)	198 (12.7%)	1415 (90.6%)	115 (7.4%)	497 (31.8%)	428 (27.4%)	45 (2.9%)
		2019	1593	28.79	284 (17.8%)***	188 (11.8%)	1516 (95.2%)***	54 (3.4%)***	586 (36.8%)**	363 (22.8%)**	62 (3.9%)
		2020	1659	29.98	169 (10.2%)***	198 (11.9%)	1562 (94.2%)	40 (2.4%)	649 (39.1%)	370 (22.3%)	123 (7.4%)***
Nevada	*March 13 - November 30*	2018	284	12.99	83 (29.2%)	153 (53.9%)	65 (22.9%)	12 (4.2%)	23 (8.1%)	91 (32.0%)	73 (25.7%)
		2019	296	13.34	103 (34.8%)	130 (43.9%)*	88 (29.7%)	12 (4.1%)	27 (9.1%)	78 (26.4%)	99 (33.4%)*
		2020	444	20.01***	109 (24.5%)**^†††^	170 (38.3%)	231 (52.0%)***	9 (2.0%)	61 (13.7%)	114 (25.7%)	160 (36.0%)
North Carolina	*March 13 - September 30*	2018	1018	17.71	366 (36.0%)	238 (23.4%)	724 (71.1%)	20 (2.0%)	320 (31.4%)	233 (22.9%)	94 (9.2%)
		2019	1014	17.47	320 (31.6%)*	194 (19.1%)*	770 (75.9%)*	21 (2.1%)	348 (34.3%)	221 (21.8%)	129 (12.7%)*
		2020	1323	22.79***	273 (20.6%)***	187 (14.1%)**	1151 (87.0%)***	10 (0.8%)**	404 (30.5%)	206 (15.6%)***	227 (17.2%)**
Rhode Island	*March 13 - November 30*	2018	207	27.17	14 (6.8%)	49 (23.7%)	167 (80.7%)	10 (4.8%)	88 (42.5%)	35 (16.9%)	5 (2.4%)
		2019	179	23.45	12 (6.7%)	28 (15.6%)*	159 (88.8%)*	9 (5.0%)	76 (42.5%)	24 (13.4%)	14 (7.8%)*
		2020	232	30.39*	1 (0.4%)***	28 (12.1%)	207 (89.2%)	6 (2.6%)	109 (47.0%)	30 (12.9%)	22 (9.5%)
Utah^c^	*March 13 - December 31*	2018	492	19.32							
		2019	454	17.58							
		2020	514	19.90							
Virginia	*March 13 - September 1*	2018	523	12.95	243 (46.5%)	96 (18.4%)	370 (70.7%)	8 (1.5%)	162 (31.0%)	67 (12.8%)	32 (6.1%)
		2019	522	12.90	226 (43.3%)	103 (19.7%)	403 (77.2%)*	4 (0.8%)	130 (24.9%)*	57 (10.9%)	46 (8.8%)
		2020	868	21.46***	250 (28.8%)***	122 (14.1%)**	781 (90.0%)***^†††^	3 (0.3%)	190 (21.9%)	74 (8.5%)	154 (17.7%)***
Wyoming	*March 13 - October 23*	2018	27	7.58	3 (11.1%)	18 (66.7%)	6 (22.2%)	2 (7.4%)	0 (0.0%)	5 (18.5%)	5 (18.5%)
		2019	22	6.17	7 (31.8%)	14 (63.6%)	7 (31.8%)	2 (9.1%)	1 (4.5%)	1 (4.5%)	4 (18.2%)
		2020	35	9.81	10 (28.6%)	14 (40.0%)	17 (48.6%)	2 (5.7%)	1 (2.9%)	8 (22.9%)	8 (22.9%)

*p < 0.05, **p < 0.01, ***p < 0.001 compared to previous year; ^†^*p* < 0.05, ^††^*p* < 0.01, ^†††^*p* < 0.001 for joinpoint regression model with 1 joinpoint (i.e. new trend). ^a^Multiple substances may be present in each death so proportions do not sum to 100%. P-values computed using Chi-squared test for independence on the proportion of deaths involving each substance vs. deaths not involving that substance; ^b^Indiana vital statistics data only include month of death, not date, so comparisons are made between monthly rates; ^c^Utah vital statistics data did not include any ICD-10 codes.

10 codes.

**Figure 1.**
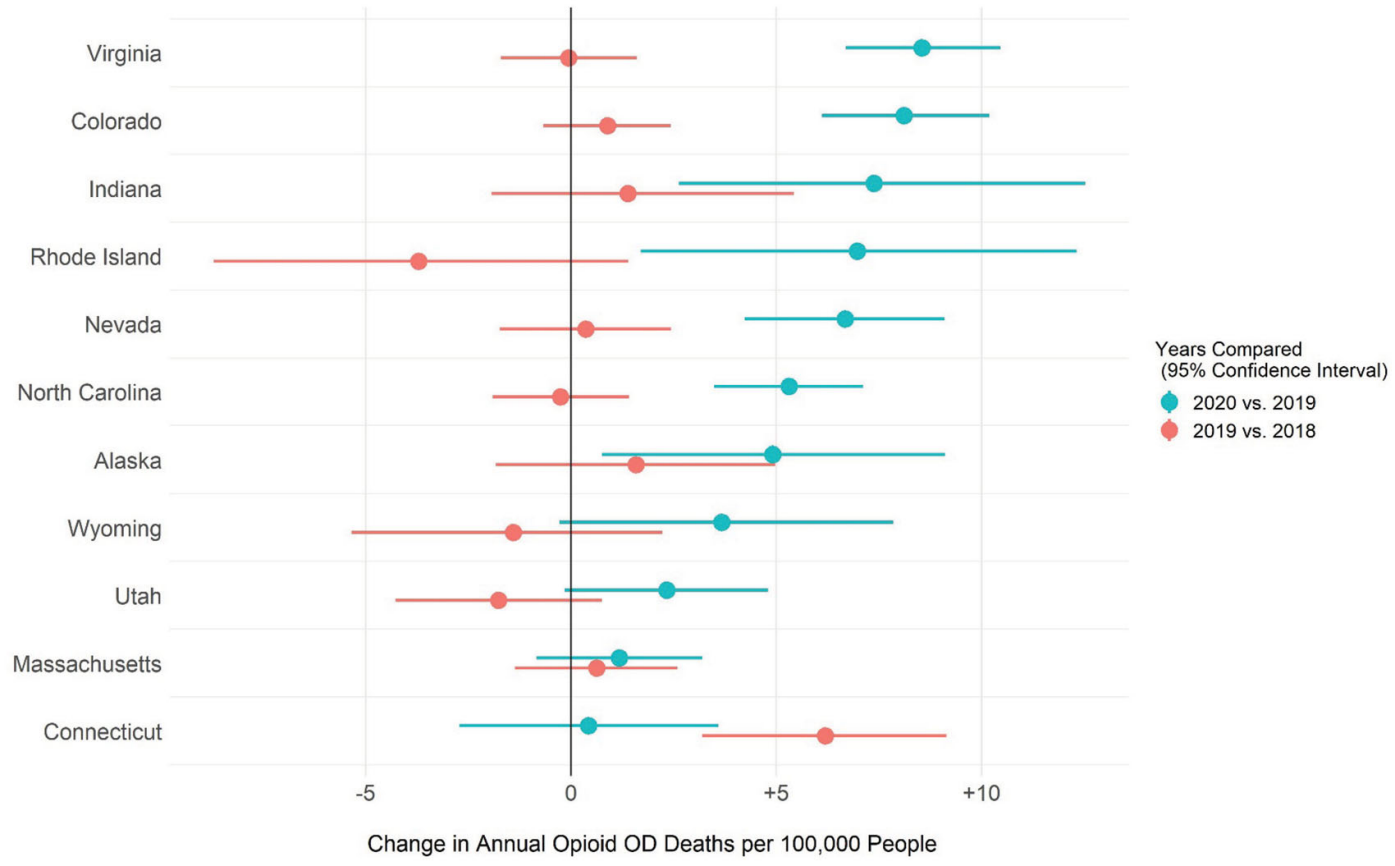
Changes in annual opioid overdose deaths per 100,000 people between 2020 vs. 2019 and 2019 vs. 2018 by state.

Figure 1 illustrates year-over-year changes in annual overdose deaths per 100,000 people.

### 3.2. Presence of opioids

There was only one opioid overdose death involving opium (T40.0) across all states and all analysis periods, so we have excluded it from our analysis. Comparing 2020 vs. 2019, the proportion of heroin-involved opioid overdose deaths was significantly reduced in Alaska (30.1% vs. 59.6%, *p =* .001), Colorado (22.0% vs. 33.0%, *p <* .001), Connecticut (15.6% vs. 30.1%, *p <* .001), Indiana (11.3% vs. 27.4%, *p <* .001), Massachusetts (10.2% vs. 17.8%, *p <* .001), Nevada (24.5% vs. 34.8%, *p* = .002), North Carolina (20.6% vs. 31.6%, *p <* .001), Rhode Island (0.4% vs. 6.7%, *p <* .001), and Virginia (28.8% vs. 43.3%, *p* < .001). Compared to 2019 vs. 2018, the results of our joinpoint regression indicate that these trends were new in Connecticut (*p* < .001) and Nevada (*p* < .001), and continuing previous trends in Alaska, Colorado, Indiana, Massachusetts, North Carolina, and Rhode Island.

Comparing 2020 to 2019, the proportion of natural and semi-synthetic opioid-involved overdose deaths was significantly reduced in Colorado (26.4% vs. 38.5%, *p <* .001), North Carolina (14.1% vs. 19.1%, *p =* .001), and Virginia (14.1% vs. 19.7%, *p* = .005). Compared to 2019 vs. 2018, joinpoint regression indicates that all shifts were continuing previous trends.

Comparing 2020 vs. 2019, the proportion of synthetic opioids among opioid-related overdose deaths significantly increased in Alaska (60.3% vs. 25.5%, *p <* .001), Colorado (59.8% vs. 41.4%, *p <* .001), Connecticut (91.5% vs. 88.3%, *p =* .019), Indiana (87.8% vs. 68.8%, *p <* .001), Nevada (52.0% vs. 29.7%, *p* < .001), North Carolina (87.0% vs. 75.9%, *p <* .001), and Virginia (90.0% vs. 77.2%, *p* < .001). Compared to 2019 vs. 2018, the significant increases in Alaska (*p* < .001), Indiana (*p* < .001), and Virginia (*p* < .001) signal new trends.

### 3.3. Presence of other psychoactive substances

Comparing 2020 vs. 2019, there has been a significant increase in the proportion of opioid-related overdose deaths involving cocaine in Alaska (13.7% vs. 2.1%, *p* = .032) and Colorado (19.1% vs. 13.2%, *p* = .037), decrease in benzodiazepines in Indiana (13.6% vs. 19.0%, *p* = .020) and North Carolina (15.6% vs. 21.8%, *p* < .001), and increase in psychostimulants in Massachusetts (7.4% vs. 3.9%, *p* < .001), North Carolina (17.2% vs. 12.7%, *p* = .003), and Virginia (17.7% vs. 8.8%, *p* < .001). Compared to 2019 vs. 2018, our joinpoint regression analysis indicates that the shift in deaths involving cocaine were new in Alaska (*p* < .001), and all other shifts were continuations of previous trends in Colorado, Massachusetts, North Carolina, and Virginia.

### 3.4. Decedent demographics

Our analysis of decedent demographics is summarised in Supplementary Tables S1-S3. Comparing 2020 vs. 2019, Colorado (overall *p =* .008) and Indiana (overall *p =* .013) experienced significant shifts in decedent sex, driven by an increase in the proportion of male decedents (70.5% vs. 61.2%, *p =* .017 in Colorado, and 70.0% vs. 62.5%, *p =* .026 in Indiana). Compared to 2019 vs. 2018, these shifts were new in Indiana (*p* < .001). Comparing 2020 vs. 2019, Massachusetts (overall *p* < .001), North Carolina (overall *p <* .001), and Virginia (overall *p* = .043) also witnessed shifts in decedent race. In Massachusetts, these shifts are related to a significant increase in the proportion of Asian non-Hispanic (1.0% vs. 0.8%, *p* = .002) and Black non-Hispanic (9.5% vs. 6.6%, *p* = .009) decedents, and an increase in the proportion of White non-Hispanic (73.8% vs. 78.3%, *p* = .009) decedents. In North Carolina, these shifts were due to a decrease in the proportion of Native American/Alaska Native/Other non-Hispanic decedents (0% vs. 2.0%, *p <* .001) and White non-Hispanic decedents (75.5% vs. 81.1%, *p =* .004), plus an increase in the proportion of Hispanic (4.4% vs. 2.6%, *p =* .048) decedents and decedents of unknown race (4.7% vs. 0.8%, *p <* .001). In Virginia, these shifts were due to an increase in the proportion of Black non-Hispanic (26.6% vs. 19.9%, *p* = .028) decedents and decrease in White non-Hispanic decedents (67.4% vs. 74.9%, *p* = .028). Only the shift in decedents of unknown race was new compared to 2019 vs. 2018 (*p* < .001). Finally, comparing 2020 vs. 2019, there were shifts in the age of decedents in Colorado (overall *p* = .016) and Nevada (overall *p* < .001). In Colorado, these shifts may have been driven by decrease in decedents aged 70–79 (1.0% vs. 4.4%, *p* = .044). In Nevada, these shifts might be owed to an increase in decedents aged 10–19 (5.4% vs. 0.7%, *p* = .009) and 20–29 (27.0% vs. 16.6%, *p* = .009). All of these shifts are continuing trends.

## 4. Discussion

cThe COVID-19 pandemic has complicated public health efforts to control the opioid crisis. This analysis extends previous single-city/state reports on opioid overdose since the onset of COVID-19, and provides more granular analyses compared to CDC’s provisional estimates. Critically, this research highlights new and continuing trends in substance use patterns and demographic characteristics among opioid overdose decedents across eleven states during the pandemic.

Previous research [3–8] and reports [12] foreshadowed rising opioid overdose death rates across the U.S. since March 2020. Our results show increases in opioid overdose death rates in several states, though all of these increases appear to be continuations of pre-existing trends. While disentangling the exact causes is challenging, these rising overdose deaths could potentially be attributed to any combination of COVID-19 related stress and isolation, limited access to in-person harm reduction services or treatment, and increasing fentanyl in the illicit drug supply [18,19]. Notably, variations in overdose deaths may partially result from the variation in states’ responses to the COVID-19 pandemic with regards to ensuring access to treatment services, and more generally with regards to public health measures such as social distancing and capacity regulations [20]. Plausibly, increases in overdose deaths related to COVID-19 could have been offset by pre-existing opioid overdose trends or overdose mitigation strategies that emerged in anticipation of the pandemic's impact. Nevertheless, ascertaining the exact causes of changing trends, while important, is beyond the scope of our analysis.

Synthetic opioid-related deaths (e.g. fentanyl and fentanyl analogs) increased significantly across seven of our 11 study states – all states that, until recently, had not experienced significant rates of synthetic opioid-involved overdose deaths [21]. This increase was new in Alaska, Indiana, and Virginia while reflecting ongoing or even stabilising trends in other states. To take New England as an example, the differences in trends defy easy explanation. Fentanyl is now involved in 90% or more of deaths in these states, *and* two of them were among the states we analysed that saw no fatal opioid overdose rate increases in 2020 (Massachusetts and Connecticut). However, Rhode Island was the only state to see a statistically significant trend reversal in 2020 that, unlike other states, could not be explained by a rise in synthetic opioid overdoses. Yet, Rhode Island is a national leader in responding to the opioid overdose crisis [22]. Anomalous patterns like these point to the possibility that differential state responses to COVID-19 could be at play. For instance, perhaps proactive implementation of harm reduction services before and during the COVID-19 pandemic (e.g. in Massachusetts) may have helped to mitigate the increase of synthetic opioid-related overdose deaths.

North Carolina and Virginia, the only two states from the South that provided data, appear to be fast-approaching the situations faced in New England, as near 90% of deaths in those states in 2020 involved synthetic opioids. Indiana, the sole Midwestern state providing data, had trends most similar to the Southern states, perhaps reflecting its relative geographic proximity. Because their trends in opioid overdose death rates and fentanyl involvement are so similar, it is difficult to speculate about the role COVID-19 or its policy responses might have played, if at all. What *is* clear is that fentanyl played a role in increasing deaths. Similarly, the Mountain West states (Colorado, Nevada, Utah, Wyoming) as well as Alaska also experienced a clear shift in fentanyl and other synthetic opioid-involved overdose deaths (and, therefore, opioid overdose rates), especially in the two more populous states (Colorado and Nevada). As with other states before them, this rise in synthetic-involved deaths is coincident with a decline in heroin-involved deaths, and in some cases, natural and semi-synthetic opioids-involved deaths, in absolute numbers and as a proportion of all deaths [23], due largely to supplantation by fentanyl. Likewise, some states in our analysis saw reductions in deaths involving prescription opioids, which has occurred nationally as numbers have generally stabilised since 2010 [9].

Our analysis of the demographic characteristics of decedents varied by state. In Indiana and Colorado, there were significant increases in the proportion of male decedents since March 13, 2020. What these states have in common is they had both experience a rise in opioid overdose death rates and a rise in the fraction involving synthetic opioids. Only two other states experienced that pattern – North Carolina and Alaska. Thus, this finding might reflect the fact that fentanyl overdoses are, in general, more likely to occur among men [24].

In all states, we found increasing proportions of Black non-Hispanic decedents and decreasing proportions of White non-Hispanic decedents since March 13, 2020. Notably, these shifts were statistically significant in Massachusetts and Virginia, potentially explaining the shifts in racial demographics overall. The non-significant increasing proportions could be due to the short study duration of this research, however, the similar pattern across all states is an important signal that warrants additional analysis. There was also a statistically significant shift in racial demographics among decedents in North Carolina, but this shift was not attributed to the increase in Black non-Hispanic decedents. This trend towards increasing deaths among Black non-Hispanic people necessitates further research to identify the cause, including potentially compounding effects of COVID-19 on rates of synthetic opioid-related deaths among Black people [25], which may be further exacerbated by the differential impacts of COVID-19 to factors including employment, housing, and access to health care and other services [26,27]. However, Black overdose death rates were increasing even prior to the COVID-19 pandemic, and could reflect shifts in the drug supply that are only just now beginning to affect Black people who use drugs [25].

We identified shifts in decedent age demographics in both Colorado and Nevada. Notably, the shift in Nevada may be driven by increases in the proportion of opioid overdose deaths among people aged 10–29. Prior regional analysis using data from 2009–2018 has identified increasing rates of opioid-related overdose mortality among young people (aged 15–34) in states east of the Mississippi River [28]. Our findings may reflect the migration of this trend in western states, or may be reflective of a larger nationwide trend of increasing overdose rates among young people [29].

Any of the increases in overdoses could potentially be related to financial impacts of COVID-19, as previous research has drawn associations between increased opioid overdose rates during times of increased financial hardship and unemployment [30,31]. However, additional analyses would be needed to confirm any causality.

Our study was limited by several data challenges [32]. Primarily, delays from each state’s death certification process resulted in varying analysis periods – preventing inter-state comparisons. Moreover, we received data only from 14 states out of 50, and District of Columbia. Of these states, data from Maryland, Mississippi, and Ohio only provided data until the end of 2019 and were thus excluded from this study. To this end, our analysis largely included states concentrated in New England and the Mountain West of the United States, though they do reflect a wide variety of experiences with both the opioid overdose crisis and COVID-19. Given state-by-state differences in opioid overdose prevention programs and COVID-19 response, this small sample of states precluded us from generating broader insights on the nature of the intersecting opioid and COVID-19 crises. Overall, these limitations affect all analyses which utilise fatal opioid overdose data and highlight the need to collectively improve data collection and reporting infrastructure.

## 5. Conclusions

This research analysed changing trends in opioid overdose deaths since the onset of COVID-19 across 11 states. To this end, various health policy researchers and organisations such as the CDC [33] and American Medical Association [34] have provided recommendations to mitigate the effects of the COVID-19 pandemic on the opioid overdose crisis. Our findings highlight new trends which can further inform each state’s response to these intersecting crises. Moreover, the trends uncovered in this analysis signal potential trends that may emerge for states with data that remain to be analysed. Accordingly, future research should expand the scope of this study as more data become available and consider a wider collection of states.

## Supplementary Material

Supplemental Material

## Data Availability

The data that support the findings of this study are available on request from the corresponding author, MSJ. Some parts of the data are not publicly available due to data agreements with states’ public health departments; however, they can be received from the states after filling out their data agreement forms.
