# Supplementary material for: Opioid overdose decedent characteristics during COVID-19
Source: Ann Med. 2022 Apr 25;54(1):1081–8. doi: 10.1080/07853890.2022.2067350 (PMC9045762; doi:10.1080/07853890.2022.2067350)
Supplement: Supplemental Material [file IANN_A_2067350_SM9867.docx]

**Online Supplementary Materials for** **Opioid Overdose Decedent Characteristics during COVID-19**

**Supplementary Table S1. Analysis of sex among opioid-related overdose decedents, 2018-2020**

| **State** | **Analysis Period** | **Year** |  | **Sex** | | |
| --- | --- | --- | --- | --- | --- | --- |
|  |  |  | *Overall* | *Female* | *Male* | *Unknown* |
| **Alaska** | *March 13 - November 30* | 2018 |  | 20 (51.3%) | 19 (48.7%) |  |
|  |  | 2019 | * | 12 (25.5%) | 35 (74.5%) |  |
|  |  | 2020 |  | 28 (38.4%) | 45 (61.6%) |  |
| **Colorado** | *March 13 - August 30* | 2018 |  | 91 (37.0%) | 155 (63.0%) |  |
|  |  | 2019 |  | 106 (38.8%) | 167 (61.2%) |  |
|  |  | 2020 | ** | 145 (29.5%)† | 347 (70.5%)† |  |
| **Connecticut** | *March 13 - December 31* | 2018 |  | 188 (24.0%) | 594 (76.0%) |  |
|  |  | 2019 |  | 231 (24.1%) | 727 (75.9%) |  |
|  |  | 2020 |  | 242 (25.0%) | 727 (75.0%) |  |
| **Indiana** | *March 1 - June 30* | 2018 |  | 137 (35.6%) | 248 (64.4%) |  |
|  |  | 2019 |  | 156 (37.5%) | 260 (62.5%) |  |
|  |  | 2020 | * | 175 (30.0%)†‡‡‡ | 408 (70.0%)†‡‡‡ |  |
| **Massachusetts** | *March 13 - December 31* | 2018 |  | 438 (28.1%) | 1123 (71.9%) |  |
|  |  | 2019 | * | 393 (24.7%) | 1200 (75.3%) |  |
|  |  | 2020 |  | 439 (26.5%) | 1220 (73.5%) |  |
| **Nevada** | *March 13 - November 30* | 2018 |  | 97 (34.2%) | 187 (65.8%) |  |
|  |  | 2019 |  | 101 (34.1%) | 195 (65.9%) |  |
|  |  | 2020 |  | 154 (34.7%) | 290 (65.3%) |  |
| **North Carolina** | *March 13 - September 30* | 2018 |  | 347 (34.1%) | 671 (65.9%) | 0 (0.0%) |
|  |  | 2019 |  | 323 (31.9%) | 691 (68.1%) | 0 (0.0%) |
|  |  | 2020 |  | 389 (29.4%) | 933 (70.5%) | 1 (0.1%) |
| **Rhode Island** | *March 13 - November 30* | 2018 |  | 50 (24.2%) | 157 (75.8%) |  |
|  |  | 2019 |  | 46 (25.7%) | 133 (74.3%) |  |
|  |  | 2020 |  | 51 (22.0%) | 181 (78.0%) |  |
| **Utah** | *March 13 - December 31* | 2018 |  | 196 (39.8%) | 296 (60.2%) |  |
|  |  | 2019 |  | 172 (37.9%) | 282 (62.1%) |  |
|  |  | 2020 |  | 202 (39.3%) | 312 (60.7%) |  |
| **Virginia** | *March 13 - September 1* | 2018 |  | 151 (28.9%) | 372 (71.1%) |  |
|  |  | 2019 |  | 177 (33.9%) | 345 (66.1%) |  |
|  |  | 2020 |  | 282 (32.5%) | 586 (67.5%) |  |
| **Wyoming** | *March 13 - October 23* | 2018 |  | 14 (51.9%) | 13 (48.1%) |  |
|  |  | 2019 |  | 8 (36.4%) | 14 (63.6%) |  |
|  |  | 2020 |  | 10 (28.6%) | 25 (71.4%) |  |

*P<0.05, **P<0.01, ***P<0.001 based on Chi-squared test for independence; †P<0.05, ††P<0.01, †††P<0.001 based on post-hoc Chi-squared test on expected residuals with Benjamini-Hochberg correction for multiple comparisons; ‡P<0.05, ‡‡P<0.01, ‡‡‡P<0.001 for joinpoint regression model with 1 joinpoint (i.e., new trend); P-values shown in 2019 row are compared to 2018 and values shown in 2020 row are compared to 2019 during the same analysis period.

**Supplementary Table S2. Analysis of race among opioid-related overdose decedents, 2018-2020**

| **State** | **Analysis Period** | **Year** |  | **Race and Ethnicity Category** | | | | | |
| --- | --- | --- | --- | --- | --- | --- | --- | --- | --- |
|  |  |  | *Overall* | *Native American/ Alaska Native/ Other NH* | *Asian NH* | *Black NH* | *Hispanic* | *Unknown* | *White NH* |
| **Alaska** | *March 13 - November 30* | **2018** |  | 4 (10.3%) | 0 (0.0%) | 1 (2.6%) | 0 (0.0%) | 0 (0.0%) | 34 (87.2%) |
|  |  | **2019** |  | 8 (17.0%) | 1 (2.1%) | 1 (2.1%) | 0 (0.0%) | 2 (4.3%) | 35 (74.5%) |
|  |  | **2020** |  | 19 (26.0%) | 0 (0.0%) | 6 (8.2%) | 4 (5.5%) | 2 (2.7%) | 42 (57.5%) |
| **Colorado** | *March 13 - August 30* | **2018** |  | 2 (0.8%) | 1 (0.4%) | 9 (3.7%) | 71 (28.9%) | 2 (0.8%) | 161 (65.4%) |
|  |  | **2019** |  | 1 (0.4%) | 6 (2.2%) | 15 (5.5%) | 60 (22.0%) | 1 (0.4%) | 190 (69.6%) |
|  |  | **2020** |  | 9 (1.8%) | 7 (1.4%) | 36 (7.3%) | 140 (28.5%) | 2 (0.4%) | 298 (60.6%) |
| **Connecticut** | *March 13 - December 31* | **2018** |  | 4 (0.5%) | 5 (0.6%) | 76 (9.7%) | 102 (13.0%) | 0 (0.0%) | 595 (76.1%) |
|  |  | **2019** |  | 5 (0.5%) | 6 (0.6%) | 103 (10.8%) | 168 (17.5%) | 1 (0.1%) | 675 (70.5%) |
|  |  | **2020** |  | 7 (0.7%) | 4 (0.4%) | 116 (12.0%) | 173 (17.9%) | 2 (0.2%) | 667 (68.8%) |
| **Indiana** | *March 1 - June 30* | **2018** |  | 0 (0.0%) | 1 (0.3%) | 40 (10.4%) | 11 (2.9%) | 1 (0.3%) | 332 (86.2%) |
|  |  | **2019** |  | 0 (0.0%) | 0 (0.0%) | 48 (11.5%) | 8 (1.9%) | 1 (0.2%) | 359 (86.3%) |
|  |  | **2020** |  | 0 (0.0%) | 3 (0.5%) | 78 (13.4%) | 17 (2.9%) | 0 (0.0%) | 485 (83.2%) |
| **Massachusetts** | *March 13 - December 31* | **2018** |  | 27 (1.7%) | 16 (1.0%) | 64 (4.1%) | 191 (12.2%) | 3 (0.2%) | 1260 (80.7%) |
|  |  | **2019** | * | 24 (1.5%) | 13 (0.8%) | 105 (6.6%)† | 203 (12.7%) | 0 (0.0%) | 1248 (78.3%) |
|  |  | **2020** | *** | 27 (1.6%) | 16 (1.0%)†† | 157 (9.5%)†† | 234 (14.1%) | 1 (0.1%) | 1224 (73.8%)†† |
| **Nevada** | *March 13 - November 30* | **2018** |  | 3 (1.1%) | 7 (2.5%) | 25 (8.8%) | 23 (8.1%) | 2 (0.7%) | 224 (78.9%) |
|  |  | **2019** |  | 6 (2.0%) | 7 (2.4%) | 28 (9.5%) | 39 (13.2%) | 2 (0.7%) | 214 (72.3%) |
|  |  | **2020** |  | 2 (0.5%) | 9 (2.0%) | 60 (13.5%) | 80 (18.0%) | 4 (0.9%) | 289 (65.1%) |
| **North Carolina** | *March 13 - September 30* | **2018** |  | 14 (1.4%) | 0 (0.0%) | 121 (11.9%) | 25 (2.5%) | 9 (0.9%) | 849 (83.4%) |
|  |  | **2019** |  | 20 (2.0%) | 0 (0.0%) | 138 (13.6%) | 26 (2.6%) | 8 (0.8%) | 822 (81.1%) |
|  |  | **2020** | *** | 0 (0.0%)††† | 0 (0.0%) | 204 (15.4%) | 58 (4.4%)† | 62 (4.7%)†††‡‡‡ | 999 (75.5%)†† |
| **Rhode Island** | *March 13 - November 30* | **2018** |  | 20 (9.7%) | 1 (0.5%) | 8 (3.9%) | 19 (9.2%) | 3 (1.4%) | 156 (75.4%) |
|  |  | **2019** |  | 10 (5.6%) | 1 (0.6%) | 12 (6.7%) | 24 (13.4%) | 2 (1.1%) | 130 (72.6%) |
|  |  | **2020** |  | 10 (4.3%) | 0 (0.0%) | 21 (9.1%) | 35 (15.1%) | 2 (0.9%) | 164 (70.7%) |
| **Utah** | *March 13 - December 31* | **2018** |  | 8 (1.6%) | 9 (1.8%) | 7 (1.4%) | 47 (9.6%) | 0 (0.0%) | 421 (85.6%) |
|  |  | **2019** |  | 10 (2.2%) | 2 (0.4%) | 5 (1.1%) | 44 (9.7%) | 2 (0.4%) | 391 (86.1%) |
|  |  | **2020** |  | 10 (1.9%) | 8 (1.6%) | 12 (2.3%) | 60 (11.7%) | 2 (0.4%) | 422 (82.1%) |
| **Virginia** | *March 13 - September 1* | **2018** |  | 1 (0.2%) | 4 (0.8%) | 110 (21.0%) | 22 (4.2%) | 0 (0.0%) | 386 (73.8%) |
|  |  | **2019** |  | 3 (0.6%) | 2 (0.4%) | 104 (19.9%) | 21 (4.0%) | 1 (0.2%) | 391 (74.9%) |
|  |  | **2020** | * | 4 (0.5%) | 6 (0.7%) | 231 (26.6%)† | 35 (4.0%) | 7 (0.8%) | 585 (67.4%)† |
| **Wyoming** | *March 13 - October 23* | **2018** |  | 1 (3.7%) | 0 (0.0%) | 0 (0.0%) | 0 (0.0%) | 0 (0.0%) | 26 (96.3%) |
|  |  | **2019** |  | 0 (0.0%) | 0 (0.0%) | 0 (0.0%) | 0 (0.0%) | 0 (0.0%) | 22 (100.0%) |
|  |  | **2020** |  | 2 (5.7%) | 1 (2.9%) | 1 (2.9%) | 0 (0.0%) | 0 (0.0%) | 31 (88.6%) |

NH=Non-Hispanic; *P<0.05, **P<0.01, ***P<0.001 based on Chi-squared test for independence; †P<0.05, ††P<0.01, †††P<0.001 based on post-hoc Chi-squared test on expected residuals with Benjamini-Hochberg correction for multiple comparisons; ‡P<0.05, ‡‡P<0.01, ‡‡‡P<0.001 for joinpoint regression model with 1 joinpoint (i.e., new trend); P-values shown in 2019 row are compared to 2018 and values shown in 2020 row are compared to 2019 during the same analysis period.

**Supplementary Table S3. Analysis of age among opioid-related overdose decedents, 2018-2020**

| **State** | **Analysis Period** | **Year** |  | **Age Group** | | | | | | | | |
| --- | --- | --- | --- | --- | --- | --- | --- | --- | --- | --- | --- | --- |
|  |  |  | *Overall* | *0-9* | *10-19* | *20-29* | *30-39* | *40-49* | *50-59* | *60-69* | *70-79* | *80+* |
| **Alaska** | *March 13 - November 30* | 2018 |  | 0 (0.0%) | 0 (0.0%) | 7 (17.9%) | 8 (20.5%) | 5 (12.8%) | 13 (33.3%) | 6 (15.4%) | 0 (0.0%) | 0 (0.0%) |
|  |  | 2019 | * | 1 (2.1%) | 0 (0.0%) | 10 (21.3%) | 20 (42.6%) | 8 (17.0%) | 4 (8.5%) | 4 (8.5%) | 0 (0.0%) | 0 (0.0%) |
|  |  | 2020 |  | 0 (0.0%) | 3 (4.1%) | 12 (16.4%) | 25 (34.2%) | 14 (19.2%) | 11 (15.1%) | 7 (9.6%) | 1 (1.4%) | 0 (0.0%) |
| **Colorado** | *March 13 - August 30* | 2018 |  | 0 (0.0%) | 6 (2.4%) | 54 (22.0%) | 68 (27.6%) | 41 (16.7%) | 47 (19.1%) | 26 (10.6%) | 3 (1.2%) | 1 (0.4%) |
|  |  | 2019 |  | 1 (0.4%) | 8 (2.9%) | 61 (22.3%) | 76 (27.8%) | 44 (16.1%) | 52 (19.0%) | 17 (6.2%) | 12 (4.4%) | 2 (0.7%) |
|  |  | 2020 | * | 0 (0.0%) | 20 (4.1%) | 128 (26.1%) | 147 (29.9%) | 82 (16.7%) | 66 (13.4%) | 42 (8.6%) | 5 (1.0%)* | 1 (0.2%) |
| **Connecticut** | *March 13 - December 31* | 2018 |  | 0 (0.0%) | 7 (0.9%) | 135 (17.3%) | 218 (27.9%) | 181 (23.1%) | 168 (21.5%) | 66 (8.4%) | 6 (0.8%) | 1 (0.1%) |
|  |  | 2019 |  | 0 (0.0%) | 3 (0.3%) | 150 (15.7%) | 273 (28.5%) | 203 (21.2%) | 215 (22.4%) | 100 (10.4%) | 12 (1.3%) | 2 (0.2%) |
|  |  | 2020 |  | 1 (0.1%) | 9 (0.9%) | 149 (15.4%) | 267 (27.6%) | 193 (19.9%) | 229 (23.6%) | 112 (11.6%) | 7 (0.7%) | 2 (0.2%) |
| **Indiana** | *March 1 - June 30* | 2018 |  | 0 (0.0%) | 2 (0.5%) | 99 (25.7%) | 114 (29.6%) | 81 (21.0%) | 66 (17.1%) | 23 (6.0%) | 0 (0.0%) | 0 (0.0%) |
|  |  | 2019 |  | 1 (0.2%) | 3 (0.7%) | 96 (23.1%) | 139 (33.4%) | 82 (19.7%) | 60 (14.4%) | 29 (7.0%) | 4 (1.0%) | 2 (0.5%) |
|  |  | 2020 |  | 0 (0.0%) | 5 (0.9%) | 127 (21.8%) | 209 (35.8%) | 127 (21.8%) | 74 (12.7%) | 38 (6.5%) | 2 (0.3%) | 1 (0.2%) |
| **Massachusetts** | *March 13 - December 31* | 2018 |  | 0 (0.0%) | 9 (0.6%) | 291 (18.6%) | 491 (31.5%) | 355 (22.7%) | 289 (18.5%) | 111 (7.1%) | 11 (0.7%) | 4 (0.3%) |
|  |  | 2019 |  | 0 (0.0%) | 15 (0.9%) | 277 (17.4%) | 490 (30.8%) | 344 (21.6%) | 340 (21.3%) | 114 (7.2%) | 11 (0.7%) | 2 (0.1%) |
|  |  | 2020 |  | 1 (0.1%) | 8 (0.5%) | 248 (14.9%) | 500 (30.1%) | 399 (24.1%) | 334 (20.1%) | 147 (8.9%) | 17 (1.0%) | 5 (0.3%) |
| **Nevada** | *March 13 - November 30* | 2018 |  | 0 (0.0%) | 3 (1.1%) | 37 (13.0%) | 58 (20.4%) | 49 (17.3%) | 70 (24.6%) | 46 (16.2%) | 19 (6.7%) | 2 (0.7%) |
|  |  | 2019 |  | 1 (0.3%) | 2 (0.7%) | 49 (16.6%) | 64 (21.6%) | 64 (21.6%) | 63 (21.3%) | 44 (14.9%) | 8 (2.7%) | 1 (0.3%) |
|  |  | 2020 | *** | 1 (0.2%) | 24 (5.4%)†† | 120 (27.0%)†† | 96 (21.6%) | 73 (16.4%) | 73 (16.4%) | 43 (9.7%) | 11 (2.5%) | 1 (0.2%) |
| **North Carolina** | *March 13 - September 30* | 2018 |  | 2 (0.2%) | 12 (1.2%) | 234 (23.0%) | 308 (30.3%) | 211 (20.7%) | 184 (18.1%) | 57 (5.6%) | 8 (0.8%) | 2 (0.2%) |
|  |  | 2019 |  | 0 (0.0%) | 16 (1.6%) | 237 (23.4%) | 304 (30.0%) | 218 (21.5%) | 157 (15.5%) | 71 (7.0%) | 10 (1.0%) | 1 (0.1%) |
|  |  | 2020 |  | 0 (0.0%) | 18 (1.4%) | 298 (22.5%) | 429 (32.4%) | 290 (21.9%) | 196 (14.8%) | 76 (5.7%) | 13 (1.0%) | 3 (0.2%) |
| **Rhode Island** | *March 13 - November 30* | 2018 |  | 0 (0.0%) | 1 (0.5%) | 36 (17.4%) | 61 (29.5%) | 47 (22.7%) | 37 (17.9%) | 25 (12.1%) | 0 (0.0%) | 0 (0.0%) |
|  |  | 2019 |  | 0 (0.0%) | 0 (0.0%) | 32 (17.9%) | 52 (29.1%) | 36 (20.1%) | 39 (21.8%) | 19 (10.6%) | 1 (0.6%) | 0 (0.0%) |
|  |  | 2020 |  | 0 (0.0%) | 2 (0.9%) | 39 (16.8%) | 62 (26.7%) | 54 (23.3%) | 53 (22.8%) | 17 (7.3%) | 4 (1.7%) | 1 (0.4%) |
| **Utah** | *March 13 - December 31* | 2018 |  | 0 (0.0%) | 12 (2.4%) | 84 (17.1%) | 130 (26.4%) | 104 (21.1%) | 98 (19.9%) | 53 (10.8%) | 10 (2.0%) | 1 (0.2%) |
|  |  | 2019 |  | 0 (0.0%) | 10 (2.2%) | 63 (13.9%) | 113 (24.9%) | 95 (20.9%) | 99 (21.8%) | 63 (13.9%) | 8 (1.8%) | 3 (0.7%) |
|  |  | 2020 |  | 0 (0.0%) | 11 (2.1%) | 67 (13.0%) | 145 (28.2%) | 128 (24.9%) | 95 (18.5%) | 49 (9.5%) | 15 (2.9%) | 4 (0.8%) |
| **Virginia** | *March 13 - September 1* | 2018 |  | 1 (0.2%) | 10 (1.9%) | 96 (18.4%) | 154 (29.4%) | 107 (20.5%) | 112 (21.4%) | 34 (6.5%) | 8 (1.5%) | 1 (0.2%) |
|  |  | 2019 |  | 0 (0.0%) | 7 (1.3%) | 96 (18.4%) | 148 (28.4%) | 112 (21.5%) | 101 (19.3%) | 51 (9.8%) | 5 (1.0%) | 2 (0.4%) |
|  |  | 2020 |  | 0 (0.0%) | 15 (1.7%) | 163 (18.8%) | 258 (29.7%) | 199 (22.9%) | 157 (18.1%) | 67 (7.7%) | 7 (0.8%) | 2 (0.2%) |
| **Wyoming** | *March 13 - October 23* | 2018 |  | 0 (0.0%) | 0 (0.0%) | 3 (12.0%) | 7 (28.0%) | 4 (16.0%) | 5 (20.0%) | 4 (16.0%) | 2 (8.0%) | 0 (0.0%) |
|  |  | 2019 |  | 0 (0.0%) | 0 (0.0%) | 2 (9.1%) | 4 (18.2%) | 5 (22.7%) | 3 (13.6%) | 5 (22.7%) | 2 (9.1%) | 1 (4.5%) |
|  |  | 2020 |  | 0 (0.0%) | 1 (2.9%) | 8 (22.9%) | 10 (28.6%) | 5 (14.3%) | 5 (14.3%) | 6 (17.1%) | 0 (0.0%) | 0 (0.0%) |

*P<0.05, **P<0.01, ***P<0.001 based on Chi-squared test for independence; †P<0.05, ††P<0.01, †††P<0.001 based on post-hoc Chi-squared test on expected residuals with Benjamini-Hochberg correction for multiple comparisons; ‡P<0.05, ‡‡P<0.01, ‡‡‡P<0.001 for joinpoint regression model with 1 joinpoint (i.e., new trend); P-values shown in 2019 row are compared to 2018 and values shown in 2020 row are compared to 2019 during the same analysis period.
